# A comparative review of current international atrial fibrillation guidelines from a primary care perspective

**DOI:** 10.51866/cpg.872

**Published:** 2025-06-07

**Authors:** Jazlan Jamaluddin, Siok Fuang Liong, Hooi Chin Beh, Karleen Chong, Jin Ian Choong, Ahmad Firdaus Zakaria, Nik Aminah Nik Abdul Kadir

**Affiliations:** 1 MD, MMed (Fam Med), Department of Primary Care Medicine, Faculty of Medicine, Universiti Malaya, Kuala Lumpur, Malaysia. Email: jazlan@um.edu.my; 2 MD, MFamMed, Department of Primary Care Medicine, Faculty of Medicine, Universiti Malaya, Kuala Lumpur, Malaysia. Email: karleenchong@um.edu.my; 3 MB BCh BAO, MFamMed, Department of Primary Care Medicine, Faculty of Medicine, Universiti Malaya, Kuala Lumpur, Malaysia.; 4 MBBS, MFamMed, Department of Primary Care Medicine, Faculty of Medicine, Universiti Malaya, Kuala Lumpur, Malaysia.; 5 MD, MFamMed, Department of Primary Care Medicine, Universiti Malaya Medical Centre, Jalan Profesor Diraja Ungku Aziz, Wilayah Persekutuan Kuala Lumpur, Malaysia.; 6 MBBS, MMed (Int Med), Cardiology Unit, Department of Internal Medicine, Hospital Al-Sultan Abdullah, Universiti Teknologi MARA, Bandar Puncak Alam, Selangor, Malaysia.; 7 MBBS, MMed (Fam Med), Klinik Kesihatan Ijok, Jalan 14, Ijok, Bestari Jaya, Selangor Darul Ehsan, Malaysia.

**Keywords:** Atrial fibrillation, Disease management, Primary healthcare, Guideline

## Abstract

Atrial fibrillation (AF) is the most frequent sustained arrhythmia globally with significant morbidity and mortality. A local guideline has been created to help physicians in clinical decision-making by outlining various generally accepted approaches for the diagnosis, management and prevention of AF. This guideline was published 10 years ago, and it is imperative that new guidelines based on the latest consensus be renewed. Since then, various clinical guidelines have been issued in the past 5 years to standardise the management of AF, including the American College of Cardiology, European Society of Cardiology, National Institute for Health and Care Excellence and Asia Pacific Heart Rhythm Society guidelines. This review aims to provide a comprehensive comparison of these guidelines across multiple aspects of clinical management, including management frameworks, screening, anticoagulation, stroke and bleeding risk assessments, rate and rhythm control, follow-up, comorbidity management and technology usage.

## Introduction

**Figure 1:**
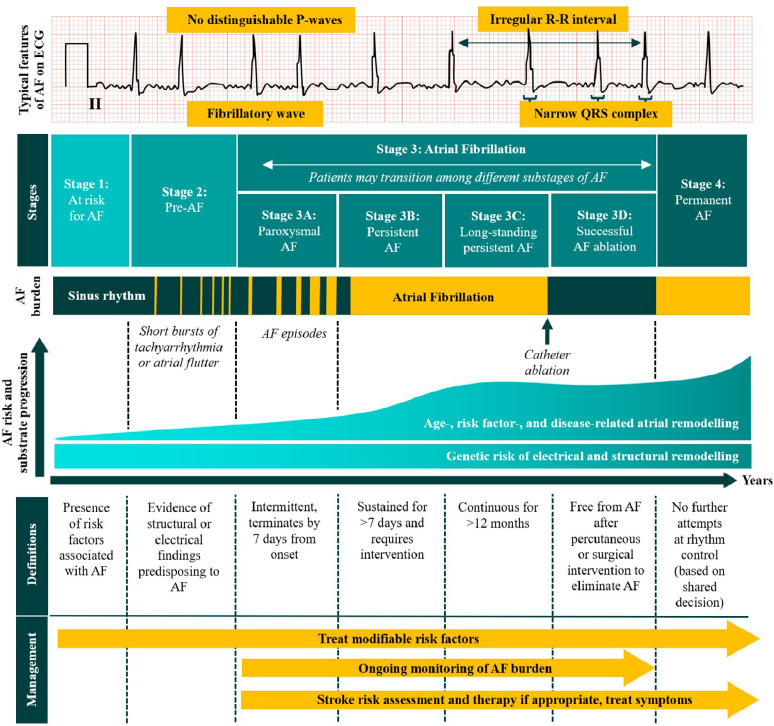
Stages and progression of AF.^2,7^ AF: atrial fibrillation

Atrial fibrillation (AF) is a supraventricular arrhythmia characterised by uncoordinated atrial activation and ineffective atrial contraction.^1,2^ It is the most prevalent sustained arrhythmia globally, affecting about 52.6 million people in 2021.^3^ The incidence of AF is rising, especially in middle-income countries, driven by ageing populations, increased survival from other cardiovascular conditions and improved detection methods. Almost one-third of older individuals are diagnosed with AF.^4^ In Malaysia, approximately 174,000 individuals are affected by AF, with about 16,000 new cases detected each year.^5^ Men generally have higher incidence rates, while ethnic and socioeconomic disparities significantly influence the prevalence and outcomes.^4,6^ There are four main stages of AF according to the duration of the disease and the effectiveness of treatment in delaying permanent AF or restoring sinus rhythm (SR) (Figure 1).^2^ Patients may be at risk of developing AF (Stage 1) due to underlying genetic factors that contribute to electrical and structural remodelling at a younger age.^2^ Other risk factors (RFs) include older age, cardiovascular diseases, hypertension, obesity, diabetes mellitus (DM), hyperthyroidism and lifestyle factors including alcohol consumption.^2^

The management of AF necessitates a comprehensive approach that considers various clinical factors and patient characteristics. Primary care plays a pivotal role in AF management through early diagnosis, timely investigation and effective treatment initiation. This setting often serves as the first point of contact for patients, making it instrumental in reducing AF-associated complications, such as stroke and heart failure (HF). A local guideline has been created to assist primary care providers in clinical decision-making by outlining various generally accepted approaches for the prevention, screening, diagnosis and management of AF.^8^ However, the guideline was published 10 years ago, and it is essential that new guidelines based on the latest consensus be updated. Recognising this, we aimed to review and summarise the most recent evidence-based guidelines for AF management in the past 5 years from major international organisations. These included the 2024 European Society of Cardiology (ESC),^4^ 2023 American College of Cardiology (ACC),^2^ 2021 United Kingdom National Institute for Health and Care Excellence (NICE)^9^ and 2021 Asia Pacific Heart Rhythm Society (APHRS) guidelines.^10,11^ For this purpose, this review will focus on the screening, diagnosis and management of AF in non-pregnant adult patients in primary care settings. By combining global recommendations with local needs, we hope to assist physicians in providing comprehensive care and improving overall health outcomes for patients with AF.

**Screening for AF**

Screening for AF can be categorised by tools, strategies and methods.^2,4,12^ 'Tools include electrocardiogram (ECG)-based screening devices, such as 12-lead ECGs, ambulatory ECG monitors, implantable devices and wearables including; smaatwa-ches ‘with ECG features. Non-ECG tools, sach as pulse palpation, photoplethysmography (PPG) and electronic blood pressure (BP) monitors with AF detection capabilities, offer sensitive and cost-effective screening options.^4,12^ However, due to their lower specificity - particularly for pulse palpation - these tools should be followed by ECG confirmation to ensure accurate diagnosis.^12,13^ Emerging wearable devices, such as fitness bands and smartwatches, show promise but currently have limited evidence for effectiveness, and studies on PPG are often of low methodological quality.^4^

Screeaing strategies include opportunistic and systematic approaches. Opportunistic scieening involves ad hoc assessments during routine healthcare visits, while systematic screening targets identified populations thrcugh methods such as mailed or yelephonic invitations. Both opportunistic and systematic strategies may use the same tools. Uptake rites are limilar for both strategies, reaching approximately 50% in primary care or large-scale studies.^10^

Screening methods include one-time or prolonged methods. One-time screening methods, including pulse palpation and 12-lead ECGs, have not shown increased detection compared to usual care.^4^ This could be due to high baseline detection in studied populations, limiting generalisability to populations with lower spontaneous detection rates. The impact of one-time screening on clinical outcomes also remains uncertain. Prolonged screening methods, including continuous or repeated intermittent monitoring, have demonstrated higher AF detection rates and small benefits in reducing combined outcomes such as mortality, stroke, embolism and severe bleeding.^14^ However, systematic reviews have shown inconsistent results regarding stroke reduction.^4^

Big data, machine learning and artificial intelligence are examples of emerging technologies that have the potential to improve AF detection, but their clinical applicability remains uncertain.^4,12^ Large-scale public screening for AF may facilitate early detection, but questions about cost-effectiveness and clinical impact persist.^2,4,13^ Any adopted screening method should consider socioeconomic and patient-specific concerns and ensure clear treatment pathways for detected cases. Screening after acute stroke and during the recovery phase with ECG-based monitoring is also recommended to improve detection rates in this high-risk population.

Recommendations from various international and regional guidelines emphasise the importance of targeted screening approaches for AF (Table 1). Overall, opportunistic screening via pulse palpation is recommended for individuals aged 65 years and above.^4,10,12,13^ Opportunistic screening using ECG or a 30-second rhythm strip may also be considered for this age group.^10,12^ Systematic screening, particularly through prolonged non-invasive ECG monitoring, such as continuous or repeated intermittent ECG, is recommended for individuals aged 75 years and above.^4,10,12^ Systematic screening for individuals at a high risk of cerebrovascular accidents (CVAs), including stroke and transient ischaemic attack, is also suggested.^10,13^ During the acute phase of CVAs, AF should be screened using pulse palpation, 12-lead ECG and inpatient continuous ECG monitoring.^10,13^ In the postacute phase, prolonged ECG monitoring through serial ECGs, ambulatory monitoring, wearable-based ECG or smartphone-based methods may be appropriate.^10^

**Table 1:** Comparison of the key recommendations of the guidelines.

Guideline (year)	ESC (2024)^4^	ACC (2023)^2^	NICE (2021)^9^	APHRS (2021)^10,11^
Screening for AF	**Opportunistic screening for patients aged ≥65 years** via routine cardiac rhythm assessment during medical visits is recommended. **Systematic screening for patients aged ≥75 years** using continuous non-invasive ECG monitoring (prolonged or repeated measurements) should be considered. Systematic screening for patients aged ≥65 years with CHA_2_DS_2_-VA RF using continuous non-invasive ECG monitoring should be considered.	No recommendations given, but discussed methods that can be employed^†^	No recommendations	**Opportunistic screening for patients aged ≥65 years** using pulse palpation or a 30-second rhythm strip is recommended. **Systematic screening for patients aged >75 years** may be considered in most countries. **Systematic screening for patients with a high risk of stroke** may be considered in most countries.
Echocardiography	**Recommended for all patients** with AF to detect heart failure and structural assessment to determine the choice of medications Limited access to TTE should not delay initiation of oral anticoagulation.	**TTE is recommended in patients with newly diagnosed AF** to assess LVEF to determine therapy modality and LA size, function and strain to predict the progression and recurrence of AF.	**TTE** is not recommended as a routine test but **should be conducted to establish a management baseline**, guide rhythm-control strategies including cardioversion, evaluate suspected heart disease and improve risk assessment for antithrombotic therapy.	**TTE is recommended** for evaluating cardiac structure and function in patients with AF.
Blood investigations	**FBC**, RP, LFT, **TFT**, HbA1c and lipid profile evaluations are recommended to detect comorbidities or risks of thrombosis and bleeding.	**FBC, TFT** and metabolic panel	No recommendation mentioned for diagnostic blood tests	No recommendation mentioned for diagnostic blood tests
Management framework	AF-CARE: **C**omorbidity and RF management, **A**void stroke and thromboembolism, **R**educe symptoms by rate and rhythm control, **E**valuation and dynamic reassessment	SOS: **S**troke risk assessment and treatment, if appropriate, **O**ptimising all modifiable RF, **S**ymptom management	Comprehensive management framework focusing on detection and diagnosis, stroke prevention and symptom management	ABC pathway: **A**void stroke with anticoagulation, **B**etter symptom management, **C**ardiovascular risk and comorbidity management
Stroke risk score	CHA_2_DS_2_-VA score	CHA_2_DS_2_-VASc score	CHA_2_DS_2_-VASc score	CHA_2_DS_2_-VASc score
Bleeding risk assessment or score	Assess for any bleeding RF. No recommendation was made on specific bleeding risk scores.	Using bleeding risk scores is not recommended due to limited evidence.	Using the ORBIT score is recommended to assess bleeding risks.	Using the HAS-BLED score is recommended for Asian patients with AF.
Rate and rhythm control	**Rate control is recommended as the initial choice for acute and stable AF**. Rhythm control can be considered in the context of shared decision-making.	Rate control is comparable to rhythm control. Thus, the use of shared decision-making with patients is recommended when deciding on therapeutic options.	**Rate control remains the first-line treatment for AF.** Rate control is preferred in acute and stable AF presenting >48 hours. Rhythm control is considered when rate control fails.	Shared and symptom-based decision-making are recommended when deciding between rate and rhythm control.
Cardioversion	**Electrical cardioversion should be initiated in patients with haemodynamic instability.** Pharmacological cardioversion is considered in patients with recent-onset AF, without underlying HFrEF, severe LVH or coronary artery disease.	**Electrical cardioversion should be initiated in patients with haemodynamic instability.** Both electrical and pharmacological cardioversion are recommended in patients with acute and stable AF but failed rate control.	**Electrical cardioversion should be initiated in patients with haemodynamic instability.** Electrical cardioversion is preferred over pharmacological cardioversion in acute and stable AF presenting >48 hours.	No recommendation was made.

Bold: Similar recommendation from different guidelines

† Screening can be considered when clinical outcomes and cost-effectiveness following screening are sufficient, consistent with the 2022 United States Preventive Services Task Force (USPSTF) guideline in that evidence is insufficient to recommend screening.

ACC: American College of Cardiology, AF: atrial fibrillation, APHRS: Asia Pacific Heart Rhythm Society, ECG: electrocardiogram, ESC: European Society of Cardiology, FBC: full blood count, HR: heart rate, LFT: liver function test, HFrEF: heart failure reduced ejection fraction, LVEF: left ventricular ejection fraction, LVH: left ventricular hypertrophy, NICE: National Institute for Health and Care Excellence, RF: risk factor, RP: renal profile, TIA: transient ischaemic attack, TFT: thyroid function test, TTE: transthoracic echocardiogram

Beyond the geriatric population, younger individuals with high-risk profiles warrant screening consideration. Those with significant RFs such as congenital heart disease, thyroid dysfunction, chronic kidney disease (CKD), obstructive sleep apnoea, obesity or a strong family history of AF should undergo targeted evaluation. Additionally, individuals with a history of cryptogenic stroke or unexplained palpitations may benefit from prolonged rhythm monitoring using ambulatory ECG or implantable loop recorders to detect subclinical AF. Regardless of the screening method adopted, it is essential to consider socioeconomic factors and patient-specific concerns. Additionally, clear treatment pathways should be established to effectively manage cases of AF detected during screening.

## Diagnosis of AF

As AF is classified as an arrhythmia, its diagnosis requires evidence of the arrhythmia’s presence. AF can present with a wide spectrum of clinical manifestations, ranging from asymptomatic to severe symptoms. Symptoms may include HF, dyspnoea, palpitations, dizziness, chest discomfort or stroke.^9^ The diagnosis of AF starts with performing pulse palpation and 12-lead ECG and ruling out valvular AF. Manual palpation for an irregular pulse remains the initial step in the clinical suspicion of AF.^9^ However, it is not diagnostic. Physical examination should also include BP measurement and auscultation to identify murmurs or evidence of HF. The definitive diagnosis of AF is established through 12-lead ECG. The key characteristics of AF in ECG include an irregularly irregular R-R interval with the absence of obvious P waves and fibrillatory atrial activity or ‘chaotic’ waves with variable morphology (Figure 1).^1^ The ESC guidelines recommend the use of either 12-lead, multiple-lead or single-lead ECG for AF diagnosis. However, 12-lead ECG remains the gold standard method for all patients diagnosed with AF, primarily to assess for coexisting structural heart disease, conduction abnormalities or ischaemic heart disease.^4^ The NICE guideline also emphasises the use of 12-lead ECG for diagnosis due to the variability in accuracy observed across multiple-lead and single-lead ECG devices (Table 1). Mobile devices utilising PPG are not recommended for the diagnosis of AF.^2^ Owing to their extended monitoring duration, implantable cardiac monitors offer the highest sensitivity for AF detection, particularly valuable in patients with cryptogenic stroke.^2^

Clinical AF is defined as AF lasting 30 seconds or more, as recorded on a body-surface ECG, and may occur with or without symptoms. In contrast, subclinical AF is defined as episodes of AF identified through continuous monitoring devices while patients remain asymptomatic.^4^ The Modified European Heart Rhythm Association symptom score is used to assess symptoms associated with AF, classifying them from none to disabling based on their impact on normal activities.^4,8^ The score considers only symptoms directly caused by AF and evaluates how they respond to the restoration of SR or effective rate control.

**Comorbidity and RF management**

All guidelines advocate for a structured approach to AF management. The ACC and NICE guidelines emphasise comprehensive management strategies that focus on prevention, diagnosis and treatment. The ESC guidelines introduce a multifaceted framework that includes comorbidity management, stroke prevention, symptom reduction and dynamic reassessment, called the AF-CARE framework. The APHRS guideline also stresses the importance of addressing comorbidities and providing patient-centred care, aligning with the broader goals of improving patient outcomes (Table 1). A central principle of these frameworks emphasises the proactive management of comorbidities and RFs as the initial therapeutic approach for all patients diagnosed with AF, irrespective of their individual thromboembolic risk profiles. Although these guidelines do not specifically outline the screening and treatment targets for each comorbidity and RF, guidelines for specific comorbidities typically include a section addressing AF, as summarised in Table 2.

**Table 2:** Addressing comorbidities and risk factors of AF.

Comorbidities	Screening modalities	Treatment and target
Hypertension^15^	Measure BP at every opportunity. Check BP for every adult at least once as part of their annual health screening and more frequently for those at risk.^*^	To reduce recurrence and progression of AF and prevent CV events ACEi or ARB may be superior in preventing recurrent AF. Target BP: 120-129/70-79 mmHg
hf^8,16^	Obtain comprehensive history of symptoms suggestive of HF. Perform echocardiography for HF screening in patients with AF. NTproBNP is helpful for ruling out HF, but not for diagnosis.	Optimise diuretics for reduced LVEF. SGLT2i for all LVEF Use beta-blockers or digoxin for rate control of AF.
Type 2 DM^1,17^	Patients with DM should be opportunistically screened at least once a year by palpating the pulse or using ECG. Consider wearable devices or Holter monitoring, although more evidence is needed.	Ensure effective glycaemic control through diet or medication.
Obesity^18^	Conduct annual screening for all adults, including BMI measurement. Body fat can be an alternative to BMI.	Aim for ≥10% of weight loss. Consider bariatric surgery for individuals with a BMI of ≥40 kg/m^2^.
Obstructive sleep apnoea	Using only symptom-based questionnaires is not recommended. Follow up with polysomnography.	Initiate CPAP to minimise apnoeic episodes.
Physical activity^19^	Use the International Physical Activity Questionnaire-Short Form.	Aim for regular moderate-to-vigorous activities, targeting 210 minutes per week.
Excess alcohol consumption^4,20^	Use the Alcohol Use Disorders Identification Test or the Cut Down, Annoyed, Guilty, Eye-Opener Questionnaire.	Limit to <3 standard drinks per week.
Smoking cessation^4^	Use the Fagerstrom test for nicotine dependence.	Strongly advise patients who smoke cigarettes to quit, and provide GDMT, including behavioural interventions and pharmacotherapy for tobacco cessation.

* *At risk: Family history of hypertension, obesity and risk factors of high BP such as dyslipidaemia, DM or smoking ACEi: angiotensin-converting enzyme inhibitor, ARB: angiotensin receptor blocker, AF: atrial fibrillation, BMI: body mass index, BP: blood pressure, CPAP: continuous positive airway pressure, CV: cardiovascular, DM: diabetes mellitus, GDMT: guideline-directed medical therapy, HF: heart failure, LVEF: left ventricular ejection fraction, NTproBNP: amino-terminal pro B-type natriuretic peptide, SBP: systolic blood pressure, SGLT2i: sodium-glucose cotransporter-2 inhibitor

**Prevention of stroke and thromboembolism**

AF increases the stroke risk by five times.^1^ Therefore, all patients are recommended to take oral anticoagulants (OACs), except for low-risk patients.^1,2,4,11^ Antiplatelet therapy (e.g. aspirin) is ineffective in preventing stroke in patients with AF and may cause harm, particularly in older adult patients. Similarly, dual antiplatelet therapy is less effective for stroke prevention than anticoagulation and increases the bleeding risk.

Clinical tools are commonly used to guide anticoagulation decisions. The CHA_2_DS_2_-VASc score is a widely used tool that more effectively risk stratifies patients with AF than the older CHADS_2_ score (Table 1).^2,10,11^ While the CHA_2_DS_2_-VA score is increasingly recognied in some guidelines, the prognostic value of female sex (Sc) remains relevant in Asian populations.^1^ Until high-quality regional data confirm its exclusion, the conventional CHA_2_DS_2_-VASc score remains widely applicable. Nevertheless, the CHA_2_DS_2_-VASc score has not been specifically validated in the Malaysian population. Newer risk scores, including the ATRIA, GARFIELD-AF and ABC stroke scores, offer modest improvements in risk discrimination compared to the CHA_2_DS_2_-VASc score but require further validation, especially regarding calibration and risk reclassification.^4^ Clinicians should also consider additional stroke and thromboembolism RFs, such as CKD, malignancy, ethnicity (e.g. Asian, Hispanic and Black) and biomarkers (e.g. B-type natriuretic peptide and troponin) and specific RFs including atrial enlargement, dyslipidaemia, smoking and obesity.^4^

OACs are categorised into direct oral anticoagulants (DOACs) and vitamin K antagonists (VKAs).^4^ A new class of OAC, namely factor XI inhibitors, is still in different stages of clinical studies and has not been recommended for widespread clinical use.^4,21^ DOACs have the advantages of reduced intracranial bleeding and no need for routine monitoring. Thus, they are now recommended by all guidelines as the preferred treatment option (Table 3). However, they are not suitable for patients with moderate-to-severe mitral stenosis or mechanical valves. Dose adjustments are required for CKD and certain drug interactions.^22^ If DOACs are not available due to prescribing restrictions or cost, referral to aid organisations or other specialities or self-purchase by patients may be necessary.

**Table 3:** DOACs available in the market.^4,22^

DOAC	Dose	Criteria for dose reduction
**Factor Xa inhibitors**		
Apixaban	Standard dose: 5 mg BD Dose reduction: 2.5 mg BD	Reduce dose if two out of three criteria are met: i. Age of ≥80 years ii. BW of ≤60 kg iii. Creatinine level of ≥133 mmol/L Can be used in advanced CKD and end-stage renal disease
Edoxaban	Standard dose: 60 mg OD Dose reduction: 30 mg OD	Reduce dose if any of the following applies: i. CrCl of 15-50 mL/min ii. BW of ≤60 kg iii. Concomitant use of erythromycin, ketoconazole, cyclosporine or dronedarone Contraindicated in cases of CrCl of <15 mL/min
Rivaroxaban	Standard dose: 20 mg OD Dose reduction: 10–15 mg OD	Reduce dose to 15 mg OD if CrCl is 15-49 mL/min. Can be used in cases of CrCl of <15 mL/min and end-stage renal disease at a dose of 10 mg OD
**Direct thrombin inhibitors**		
Dabigatran	Standard dose: 150 mg BD Dose reduction: 110 mg BD	Reduce dose if any of the following applies: i. Age of ≥80 years ii. Concomitant use of verapamil Consider reducing the dose based on an individualised decision if any of the following applies: i. Age of 75-80 years ii. CrCl of 30-50 mL/min iii. Gastritis, gastro-oesophageal reflux or oesophagitis iv. Higher risk of bleeding Contraindicated in cases of CrCl of <30 mL/min

BD: twice daily, BW: body weight, CKD: chronic kidney disease, CrCl: creatinine clearance, DOAC: direct oral anticoagulant, OD: once daily

VKAs, including the most commonly used one - warfarin, are effective in patients with moderate-to-severe mitral stenosis or mechanical valves. However, they require regular international normalised ratio (INR) monitoring (target: 2.0-3.0) and maintenance of >70% therapeutic range. Local guidelines are available to assist in the adjustment of warfarin dosages.^23^ Dietary vitamin K intake and drug interactions play a crucial role in anticoagulation control. However, it is advisable to minimise unnecessary dose adjustments to prevent unstable INR readings. Switching to DOACs is recommended for patients with poor INR control (<70%) or a high bleeding risk. Inappropriate dose reductions of DOACs should be avoided; drug interactions should be monitored; and patients should be involved in shared decision-making to improve adherence. In select cases (e.g. acute coronary syndrome), a combination of anticoagulants and antiplatelets may be prescribed. The residual stroke risk can persist despite anticoagulation due to non-AF mechanisms, poor adherence or inappropriate dosing.

Left atrial appendage occlusion, whether percutaneous or surgical, is an option for patients who are ineligible for anticoagulation. It provides stroke prevention outcomes comparable to those of warfarin, albeit with procedural risks. Post-implantation, antithrombotic therapy is often required to balance stroke prevention and bleeding risks. For subclinical AF, evidence for anticoagulation is limited despite its standard treatment in clinical AF. DOACs such as apixaban may reduce stroke or systemic embolism risks but increase bleeding risks.^24^

DOACs may be used selectively in patients with high stroke risks without major bleeding risks. Routine switching between anticoagulants is not recommended unless clinically indicated. Additionally, the management of cardiovascular RFs should be prioritised.

Managing bleeding risks is an important aspect in AF care. The use of bleeding risk scores to determine whether to initiate or withdraw anticoagulation is not recommended by most guidelines, as it may lead to under-treatment. Instead, bleeding RFs, such as hypertension and non-steroidal anti-inflammatory drug (NSAID) use, must be assessed and addressed regularly. Nevertheless, most guidelines still recommend using the HAS-BLED score to assess the risk of bleeding and identify high-risk patients (Table 1). Proton pump inhibitors may be prescribed for patients at a high risk of gastrointestinal bleeding, although evidence is limited. In cases of mild bleeding, anticoagulants may be temporarily stopped, while major bleeding may necessitate the use of specific antidotes, such as andexanet alfa for factor Xa inhibitors or idarucizumab for dabigatran or prothrombin complex concentrates. Patient education plays a vital role in managing AF. Patients should be counselled on the recognition of bleeding symptoms and the importance of adhering to prescribed medications.

**Symptom reduction by rate and rhythm control**

Most patients with AF will require treatment and/or procedures to control heart rate (HR) and restore or maintain SR outcomes to alleviate symptoms or improve overall outcomes.

*HR management*

Rate control therapy is recommended in acute care settings as the initial approach, either as a standalone strategy or in addition to rhythm control therapy to manage HR and improve AF-related symptoms. The ACC, ESC and NICE guidelines suggest a more relaxed target HR control of <110 beats per minute (bpm). Evidence indicates that this lenient approach is non-inferior to a stricter regimen (<80 bpm at rest and <110 bpm on exertion) for composite outcomes of clinical events, HF class or hospital admission.^25-27^ In contrast, the JCH and APHRS guidelines recommend individualised rate control strategies.

In acute presentations of AF, management focuses on identifying and addressing the underlying cause, such as sepsis, fluid overload or cardiogenic shock. Beta-blockers (BBs) are the first-line treatment for all left ventricular ejection fractions (LVEFs), while diltiazem or verapamil is preferred for patients with an LVEF of >40%.^28-30^ Digoxin may be used in combination therapy when indicated. In patients who are unstable haemodynamically or have severely impaired LVEF, intravenous amiodarone or digoxin can be considered.^31,32^

Long-term rate control can be maintained using BBs, verapamil, diltiazem, digoxin or a combination of therapies. The choice of drugs is individualised based on patient symptoms, existing comorbidities and possible side effects or drug interactions. Due to the risk of adverse effects, a combination of BBs with verapamil or diltiazem should only be prescribed in secondary care. Amiodarone is reserved as a last resort for long-term control because of its extensive extracardiac adverse effect profile (Table 4).

**Table 4:** Choice of agents for rate and rhythm control.

Agent	IV administration	Oral maintenance dose	Contraindication
**BBs**			
Metoprolol tartrate	2.5–5 mg bolus over 2 minutes; maximum up to 15 mg cumulative dose	25–100 mg BD	Non-selective BBs to be avoided in patients with asthma BBs are contraindicated in patients with acute heart failure and a history of severe bronchospasm.
Esmolol	500 mcg/kg IV bolus over 1 minute; followed by 50–300 mcg/kg/min	Not available	
Bisoprolol	Not available	1.25–20 mg OD	
Atenolol	Not available	25–100 mg OD	
Nebivolol	Not available	2.5–10 mg OD	
Carvedilol	Not available	3.125–50 mg BD	
Metoprolol XL (succinate)^*^	Not available	50–200 mg OD	
Landiolol^*^	100 mcg/kg IV bolus over 1 minute; followed by 10–40 mcg/kg/min	Not available	
**Non-dihydropyridine calcium channel antagonists**			
Verapamil	2.5–10 mg IV bolus over 5 minutes	40 mg BD to 480 mg (extended release) OD	Contraindicated if the left ventricular ejection fraction is <40%; adjust doses in patients with liver and kidney impairments.
Diltiazem	0.25 mg/kg IV bolus over 5 minutes, then 5–15 mg/h	60 mg TDS to 360 mg (extended release) OD	
**Digitalis glycosides**			
Digoxin	0.5 mg IV bolus (0.75–1.5 mg over 24 hours in divided doses)	0.0625–0.25 mg OD	High plasma levels are associated with adverse events. Check renal function before initiation, and adjust dose in patients with CKD.
Digitoxin^*^	0.4–0.6 mg	0.05–0.1 mg OD	
**Others**			
Amiodarone	300 mg IV diluted in 250 mL 5% dextrose over 30–60 minutes (preferably via a central venous line), followed by 900–1200 mg IV over 24 hours diluted in 500–1000 mL via a central venous line	200 mg OD after loading Loading: 200 mg TDS for 4 weeks, then 200 mg OD or less as appropriate (reduce other rate control drugs guided by the heart rate)	Contraindicated in patients with iodine sensitivity. Potential serious adverse effects (involving the lungs, eye, liver and thyroid) may occur. Consider various drug interactions.

* Not registered for use in Malaysia

BB: beta-blocker, BD: twice daily, CKD: chronic kidney disease, IV: intravenous, OD: once daily, TDS: three times daily

Highly symptomatic patients with permanent AF with at least one hospital admission for HF should be considered for atrioventricular node ablation combined with cardiac resynchronisation therapy to reduce physical limitations, symptoms, recurrent HF admission and mortality.^33,34^

*Rhythm control strategies*

The goals of rhythm control in AF are to restore and maintain SR with various therapies, including anti-arrhythmic drugs (AADs), cardioversion and ablation techniques such as percutaneous catheterisation as well as endoscopic, hybrid and open surgical approaches. The ACC, ESC and NICE guidelines provide comprehensive strategies for rhythm control, including electrical cardioversion, while the JCH and APHRS guidelines emphasize shared decision-making in selecting appropriate rhythm control methods. Rapid electrical cardioversion is recommended in patients with AF and acute or worsening haemodynamic instability. Cardioversion of AF (electrical or pharmacological) should be considered in symptomatic patients with persistent AF as part of their rhythm control approach. Although it is less effective than electrical cardioversion, pharmacological cardioversion to restore SR can be conducted electively for patients who are haemodynamically stable.

In patients with AF and acute or worsening haemodynamic instability, rapid electrical cardioversion is recommended.^35^ As part of the rhythm control approach, patients with persistent AF should be considered for either electrical or pharmacological cardioversion. For patients with AF who are haemodynamically stable, pharmacological cardioversion to revert to SR can be performed as an elective procedure, although it is less effective than electrical cardioversion.^36^ Long-term rhythm control aims to maintain SR, enhance quality of life, slow AF progression and possibly reduce morbidity related to AF episodes.^22,23^ There are many serious cardiac and extracardiac side effects related to AADs, including hypotension, proarrhythmia and organ toxicity. Thus, drug safety should determine the drug of choice, rather than efficacy. Examples of AADs are flecainide and amiodarone. Propafenone, ibutilide and vernakalant are not available in Malaysia. Patients with symptomatic persistent AF not responding to AADs should be considered for ablation procedures.

**Follow-up**

The management of AF requires adaptable care due to its dynamic nature and associated comorbidities. Regular reassessment is essential and recommended by all guidelines to adjust tailored treatment plans, address modifiable RFs and allow early detection of complications such as HF and stroke. Follow-ups are recommended at least 6 months after diagnosis and annually based on individual needs. Multidisciplinary care involving general practitioners, cardiologists, nurses and pharmacists is crucial for continuity. Flexible models, including nurse-led and technology-integrated approaches, highlight the importance of patient-specific strategies.

Cardiac imaging, particularly echocardiography, is vital for detecting heart issues and assessing AF-related changes. Advanced imaging techniques and patient-reported outcome measurement tools can help evaluate treatment effectiveness. Treatment adherence remains a challenge due to cultural, psychological and systemic barriers. Empowering patients and involving them in decision-making through tools including mobile applications and wearable devices can improve their engagement and treatment adherence. Optimising AF management requires continuous reassessment, proactive care and multidisciplinary collaboration to enhance patient outcomes and satisfaction.

**Limitations**

Most of the referenced guidelines originate from high-income countries, where resource availability differs significantly from local primary care settings. For example, the recommendation of DOACs as first-line therapy is not universally feasible, as DOACs are not widely available in public primary care clinics. Warfarin remains the mainstay anticoagulant in these settings, yet logistical constraints exist, such as limited access to same-day INR testing in most clinics, complicating timely dose adjustments. Additionally, the referral pathways from primary to secondary care may vary according to the local healthcare framework. Oral AADs are not typically initiated in primary care settings, and parenteral forms are almost exclusively used in hospitals. However, initiatives in the field are ongoing to improve their availability in public primary care settings.

**How to apply the guidelines into practice in primary care**

Early detection and timely intervention are critical, as primary care providers are often the first points of contact. Screening for AF should focus on high-risk individuals, such as those aged 65 years and older, using tools including pulse palpation and ECG. Early identification enables timely management to prevent complications. It is important to assess potentially reversible triggers, such as recent alcohol consumption, hyperthyroidism and acute pulmonary conditions. The CHA_2_DS_2_-VA score should be used for stroke risk stratification to guide anticoagulation decisions. Patients with a CHA_2_DS_2_-VA score of 1 or higher should start on DOACs unless contraindicated. Shared decision-making ensures patients understand the benefits and risks of treatment. Management must include lifestyle modifications and optimisation of comorbidities such as hypertension, DM and obesity. BBs or calcium channel blockers are preferred for rate control. Digoxin should be avoided as monotherapy in older adults. Patients requiring rhythm control or advanced interventions, such as cardioversion or catheter ablation, should be referred to cardiologists early.

Follow-ups are critical to monitor treatment efficacy, adherence and complications. Initially, monthly reviews, if possible, should be conducted to assess anticoagulation and symptom control, extending to 6-12 months once stable. Regular reassessment of stroke and bleeding risks and renal function ensures continued appropriateness of therapy. Medication reviews help mitigate interactions and bleeding risks, such as avoiding NSAIDs. Collaboration with multidisciplinary team members, including pharmacists and nurses, enhances care delivery. Leveraging technology, such as electronic health records and wearable devices, supports monitoring and prompts timely interventions. Regular audits ensure adherence to evolving guidelines. By integrating guidelines into practice, primary care providers can improve early detection, ensure effective management and reduce complications, optimising the outcomes of patients with AF.

**Case vignette**

A 65-year-old man presented for a follow-up appointment in a primary care clinic, having been diagnosed with AF, confirmed via ECG 6 months ago during an evaluation for palpitations. Transthoracic echocardiogram showed a normal LVEF and no valvular abnormalities. The patient had a history of DM, hypertension and dyslipidaemia. His haemoglobin level and thyroid, renal and liver function were all normal. The CHA_2_DS_2_-VA score was 3 (1 point each for age of ≥65 years, hypertension and DM). He agreed to receiving anticoagulation therapy with a DOAC and was started on a BB for rate control. Prior to this, his comorbid conditions had been optimised: His HbA1c level was reduced to 6.8% through adjustments to his DM regimen; his BP stabilised at 125/80 mmHg on an ACE inhibitor; and his LDL cholesterol level decreased to 1.4 mmol/L with statin therapy. Lifestyle interventions, including weight management and sodium restriction, were also initiated.

Over the past 6 months, the patient remained stable. He adhered to the DOAC therapy, with no bleeding or thromboembolic events. Symptom control was achieved with the BB, and a subsequent ECG in the clinic confirmed effective rate control. However, he intermittently used NSAIDs for knee pain. He was advised about the bleeding risks associated with NSAIDs and switched to acetaminophen as a safer alternative. He was scheduled for a follow-up in 6 months to continue monitoring his symptoms, maintain lifestyle modifications and check renal function for safe DOAC dosing and other RF optimisation, with an adjustment of overall therapy if necessary. He was advised regarding referral to a cardiologist if rhythm control becomes a priority or if his symptoms worsen.

## Conclusion

Primary care is key to detecting and managing AF early while preventing complications. This review simplifies the latest guidelines, offering practical strategies for better patient care. By using new screening tools, improving diagnosis and focusing on personalised care and risk management, primary care providers can effectively tackle the growing challenges of AF.
